# Kras promotes myeloid differentiation through Wnt/β‐catenin signaling

**DOI:** 10.1096/fba.2019-00004

**Published:** 2019-07-01

**Authors:** Noriko Yokoyama, Yeon‐Jeong Kim, Yoshio Hirabayashi, Yoko Tabe, Kenji Takamori, Hideoki Ogawa, Kazuhisa Iwabuchi

**Affiliations:** ^1^ Institute for Environmental and Gender Specific Medicine Juntendo University Graduate School of Medicine Urayasu Chiba Japan; ^2^ Laboratory for Neuronal Growth Mechanisms Riken Brain Science Institutes Saitama Japan; ^3^ Cellular Informatics Laboratory RIKEN Wako Saitama Japan; ^4^ Department of Laboratory Medicine Juntendo University School of Medicine Hospital Hongo Tokyo Japan; ^5^ Infection Control Nursing Juntendo University Graduate School of Health Care and Nursing Urayasu Chiba Japan; ^6^ Laboratory of Biochemistry Juntendo University Faculty of Health Care and Nursing Urayasu Chiba Japan

**Keywords:** GSK3β, HL‐60 cell differentiation, tumor suppressor, wild‐type Kras, Wnt/β-catenin

## Abstract

Wild‐type Kras, a small GTPase, inactivates Ras growth‐promoting signaling. However, the role of Kras in differentiation of myeloid cells remains unclear. This study showed the involvement of Kras in a novel regulatory mechanism underlying the dimethyl sulfoxide (DMSO)‐induced differentiation of human acute myeloid leukemia HL‐60 cells. Kras was found to positively regulate DMSO‐induced differentiation, with the activity of Kras increasing upon DMSO. Inhibition of Kras attenuated CD11b expression in differentiated HL‐60 cells. GSK3β, an important component of Wnt signaling, was found to be a downstream signal of Kras. Phosphorylation of GSK3β was markedly enhanced by DMSO treatment. Moreover, inhibition of GSK3β enhanced CD11b expression and triggered the accumulation in the nucleus of β‐catenin and Tcf in response to DMSO. Inhibitors of β‐catenin‐mediated pathways blocked CD11b expression, further indicating that β‐catenin is involved in the differentiation of HL‐60 cells. Elevated expression of C/EBPα and C/EBPɛ accompanied by the expression of granulocyte colony‐stimulating factor (G‐CSF) receptor was observed during differentiation. Taken together, these findings suggest that Kras engages in cross talk with the Wnt/β‐catenin pathway upon DMSO treatment of HL‐60 cells, thereby regulating the granulocytic differentiation of HL‐60 cells. These results indicate that Kras acts as a tumor suppressor during the differentiation of myeloid cells.

**Abbreviations**
AKT: protein kinase B
AML: acute myeloid leukemia
C/EBP: CCAAT/enhancer‐binding protein
DHL‐60: HL‐60 cells differentiated into neutrophilic lineage
DMSO: dimethyl sulfoxide
G‐CSF: granulocyte colony‐stimulating factor
PMCA: calcium pump pan PMCA ATPase
SDS‐PAGE: sodium dodecyl sulfate‐polyacrylamide gel electrophoresis
Tcf/Lef: T‐cell factor/lymphoid enhancer factor

## 1 INTRODUCTION

The *Ras* genes encode small GTP‐binding proteins that are involved in many cellular processes, including proliferation, differentiation, and apoptosis.^1^ Wild‐type Ras proteins have intrinsic GTPase activity, catalyzing the hydrolysis of bound GTP to GDP and thereby inactivating Ras growth‐promoting signaling. In contrast, mutant Ras is locked into the GTP‐bound state, leading to constitutive Ras signaling.^1^, ^2^ Three members of the Ras family, Hras, Kras, and Nras, were found to be activated by mutation in various human cancers.^1^

Ras isoforms are ubiquitously expressed and highly homologous, but have specific and distinct molecular functions.^1^ In acute myeloid leukemia (AML) and related myelodysplastic syndromes, the most frequently mutated genes are *N‐* and *Kras*.^3^ Despite the high frequency of *N‐* and *Kras* mutations in AML, the precise roles of *ras* oncogenes in leukemogenesis remain unclear. Kras knockout is embryonically lethal in mice, whereas Hras and Nras double knockout mice develop normally.^4^ These findings indicate that Kras is essential for normal mouse development, whereas Hras and Nras are not.^5^

Mutants of *Kras* play essential roles during malignant transformation in human cancers.^1^, ^6^, ^7^ Mutated Kras induces tumor cell migration through the activation of the MAPKs and PI3K/AKT pathways.^2^, ^8^ Kras conditional knockout mice develop profound hematopoietic defects, including splenomegaly, an expanded neutrophil compartment, and reduced B‐cell number, indicating that Kras is required for adult hematopoiesis.^9^ Hematopoietic cell‐specific deletion of Kras impaired B‐cell development, but did not affect T‐cell development,^10^ suggesting that, despite its oncogenic activity, Kras plays distinct roles in hematopoietic stem cells.

Evidence has suggested that wild‐type Kras is involved in other than oncogenic activity.^11^, ^12^, ^13^ For example, the expression of p21Ras proteins is upregulated during the differentiation of HL‐60 cells.^14^ Less is known, however, about the contribution of the Kras signaling pathway to differentiation processes. This study therefore focused on novel functions of Kras and Kras‐mediated signaling networks in the differentiation of the human acute myeloid leukemia cell line HL‐60, which is one of the most common forms of leukemia cell lines and widely used in studies of human myeloid cell differentiation.^15^ HL‐60 cells are neutrophilic promyelocytes, which can be differentiated into neutrophil‐like, monocyte‐like, or eosinophil‐like cells depending on the method of differentiation. For example, HL‐60 cells can be differentiated into granulocytic cells upon exposure to polar compounds such as dimethyl sulfoxide (DMSO).^16^ However, the detailed mechanisms underlying the myeloid differentiation of HL‐60 cells remain unclear.

Wnt signaling is involved in many cellular events, including development, proliferation, differentiation, and migration.^17^, ^18^, ^19^ Aberrant canonical or non‐canonical Wnt signaling is involved in the pathogenesis of various cancers including AML.^18^ The Wnt/β‐catenin pathway has been shown to play essential roles in regulating the proliferation, differentiation, and apoptosis of hematopoietic stem cells.^20^ However, the exact functions of the Wnt signaling pathway in leukemia have not yet been fully clarified, with various studies yielding conflicting results. The protein β‐catenin is essential to the canonical Wnt cascade, and its stability is controlled by a destruction complex consisting of β‐catenin, the adenomatous polyposis coli (APC) protein, the cytoplasmic serine/threonine kinase GSK3β, CK1, and Axin. Phosphorylation of β‐catenin by CK1 and GSK3β turns off Wnt signaling. Phosphorylated β‐catenin, in turn, is degraded through proteasomes.^21^, ^22^, ^23^ In contrast, activation of Wnt signaling results in the phosphorylation of GSK3β at Ser 9, inactivating GSK3β activity and leading to the accumulation of non‐phosphorylated β‐catenin in the cytoplasm. This accumulated β‐catenin translocates into the nucleus and interacts with the transcription factors T‐cell factor/lymphoid enhancer factor (Tcf/Lef), activating the transcription of target genes.^21^, ^22^, ^23^ Activation of Tcf/Lef signaling reduces apoptosis of normal progenitor cells.^24^

The CCAAT/enhancer‐binding protein (C/EBP) is a modular protein, containing a carboxy‐terminal leucine zipper dimerization domain, a DNA‐binding domain, and an N‐terminal activation domain. C/EBPα is highly expressed in myeloblastomas, and C/EBPα‐deficient mice fail to undergo myeloid differentiation.^25^ C/EBPα acts as a molecular switch during early hematopoietic development.^26^ Neutrophilic differentiation is dependent on C/EBPα, which, in turn, is directly regulated by Lef‐1.^27^ Thus, Lef‐1 plays a crucial role in neutrophilic granulopoiesis, and C/EBPɛ, which is expressed only in hematopoietic tissues, is especially important in normal myeloid development.^28^ C/EBPɛ is essential for the terminal differentiation and functional maturation of granulocyte progenitor cells.^29^ Functional neutrophils are absent from C/EBPɛ‐deficient mice,^29^ and treatment of HL‐60 cells with DMSO for 3 days has been reported to increase the expression of the C/EBPɛ gene.^30^

Low concentrations of DMSO remove surface water from phospholipid bilayers,^31^ suggesting that DMSO affects the fluidity of plasma membranes and activates several biological functions of cells. Although DMSO induces HL‐60 cell differentiation, the mechanisms that regulate this differentiation process have not yet been fully elucidated. β‐Catenin expression is upregulated in most AMLs, whereas treatments targeting β‐catenin are not always successful in patients with AML.^20^ Wnt signaling in hematopoiesis is complex, but the involvement of Wnt/β‐catenin signaling in leukemia stem cell developement remains unclear.^20^

This study therefore assessed the mechanism underlying the myeloid differentiation of HL‐60 cells, including the involvement of β‐catenin. HL‐60 cells were induced by DMSO to differentiate into granulocytic cells, and the signaling pathways involved in this differentiation were examined. This study found that Kras activity was upregulated and that there was cross talk between Kras and Wnt/β‐catenin signaling in DMSO‐differentiated HL‐60 cells, suggesting a novel function of Kras. Moreover, this study found that elevated expression of C/EBPα, C/EBPɛ, and G‐CSF receptor was involved in the differentiation of HL‐60 cells.

## 2 MATERIALS AND METHODS

### 2.1 Materials

DMSO, anti‐β‐catenin antibody, RPMI 1640, and pyrvinium pamoate were from Sigma‐Aldrich (St. Louis, MO). Complete protease inhibitor cocktail (Complete) was from Roche Diagnostics (Tokyo, Japan). CHIR‐99021 was from Focus Biomolecules (Plymouth Meeting, PA). PPKF115‐584 was from BioVision, Inc (Milpitas, CA). Immobilon membrane was from Millipore (Bedford, MA). The Kras inhibitor SAH‐SOS1A was from Merck (Darmstadt, Germany). Anti‐Lamin B1, anti‐C/EBPα, anti‐C/EBPɛ, anti‐calcium pump pan PMCA ATPase (PMCA), anti‐G‐CSF receptor antibodies, and AKT inhibitor (AKTi‐1/2) were from Abcam (San Francisco, CA). Anti‐GSK3β, anti‐p‐GSK3β (Ser9), and anti‐Tcf4/Tcf7L2 antibodies were from Cell Signaling Technology (Danvers, MA). Anti‐G‐CSF antibody was from R&D Systems (Minneapolis, MN). Human Kras and control siRNAs were from Dharmacon (Lafayette, CO). PE‐labeled mouse IgG1k isotype control and PE‐labeled anti‐human CD11b were from eBioscience, Inc (San Diego, CA). Nuclear Extract Kits were from Active Motif (Carlsbad, CA), and Kras Activation Assay Kits were from Cell Biolabs, Inc (San Diego, CA). The luciferase assay system was from Promega Corporation (Madison, WI). Plasmids of Super8xTOPFlash (M50) and Super8xFOPFlash (M51) were kind gifts from Dr. Craig C. Malbon (State university of New York at Stony Brook, Stony Brook, NY).

### 2.2 Cell culture and transfection

HL‐60 cells were purchased from the American Type Culture Collection (ATCC; Manassas, VA) and were maintained in RPMI 1640 medium supplemented with 10% fetal bovine serum (FBS) and penicillin/streptomycin at 37°C in 5% CO_2_. HL‐60 cells in this study were regarded as a model of AML, not acute promyelocytic leukemia (APL), because these cells were negative for promyelocytic leukemia/retinoic acid receptor alpha (PML‐RARA). HL‐60 cells were differentiated into neutrophilic lineage cells (DHL‐60) by culture in medium containing 1.3% DMSO for the indicated times. In some experiments, cells were transfected with control or Kras siRNA using an Amaxa Nucleofector Kit (Amaxa Biosystems, RONZA, Japan) according to the manufacturer's protocol, followed by further culture for 96 hours in RPMI 1640 medium supplemented with 20% FBS plus 1.3% DMSO.

### 2.3 Cellular lysates

HL‐60 and DHL‐60 cells (1 × 10^7^ cells each) were lysed with 250 μl of RIPA buffer (50 mmol/L Tris‐HCl, pH 7.2, 150 mmol/L NaCl, 5 mmol/L EDTA, 2 mmol/L sodium orthovanadate, 1% NP‐40, 0.25% sodium deoxycholate, 0.05% SDS) for 30 minutes at 4°C. The lysates were centrifuged for 10 minutes at 20 800× *g*, and 20 μl aliquots were subjected to SDS‐PAGE and analyzed by Western blotting with the indicated antibodies.

### 2.4 Fractionation of cytoplasm and plasma membrane

HL‐60 and DHL‐60 cells (1 × 10^8^ cells each) were washed twice with PBS, pretreated with 5 mmol/L isopropyl fluorophosphates (DFP) in PBS for 10 minutes, suspended in 2.5 ml of relaxation buffer (100 mmol/L KCl, 3.5 mmol/L MgCl_2_, 1 mmol/L ATP‐2Na, 10 mmol/L PIPES, pH 7.3), and homogenized by nitrogen cavitation for 20 minutes at 400 psi on ice. Each fraction was dropped into a tube containing EDTA (final concentration, 1.25 mmol/L), and the tubes were centrifuged at 450× *g* for 10 minutes. The supernatants were decanted and further centrifuged at 4900× *g* for 20 minutes at 4°C to remove granules. Plasma membrane and cytoplasm fractions were separated by centrifuging these supernatants at 70 000× *g* for 1 hour. Each precipitated membrane fraction was suspended in 150 μl of RIPA buffer containing protease inhibitor cocktail (Complete). The supernatant fractions were regarded as cytoplasm. Aliquots (20 μl) of cytoplasm and plasma membrane fractions were subjected to SDS‐PAGE and analyzed by Western blotting with the indicated antibodies.

### 2.5 Extraction of nuclear fraction

Nuclear fractions were prepared using Nuclear Extract Kits (Active Motif), according to the manufacturer's instructions. Briefly, HL‐60 and DHL‐60 cells (2 × 10^7^ cells each) were washed twice with PBS, suspended in 1 ml hypotonic buffer, and incubated on ice for 15 minutes. After detergent was added to the samples, the suspensions were centrifuged at 14 000× *g* for 30 seconds at 4°C. The pellets were resuspended in 100 μl of lysis buffer AMI‐containing protease inhibitors and 10 mmol/L DTT, and incubated on ice for 30 minutes. Suspensions were centrifuged for 10 minutes at 14 000× *g*. Supernatants were regarded as the nuclear fraction. Proteins (20 μg) were analyzed by Western blotting with the indicated antibodies.

### 2.6 Western blotting

Proteins were separated on SDS‐polyacrylamide gels and transferred to Immobilon membranes. The blots were incubated with the antibodies indicated in the figure legends. Immune complexes were detected by incubation with horseradish peroxidase (HRP)‐conjugated antibody and measurement of ECL chemiluminescence (Pierce Biotechnology, Rockland, IL). To measure the relative amount of proteins, blots were incubated with primary antibodies to the respective phosphorylated or target proteins and then with HRP‐conjugated secondary antibodies. Membranes were stripped of antibodies by incubation with stripping buffer (62.5 mmol/L Tris‐HCl, pH 6.8, 100 mmol/L β‐mercaptoethanol, 2% SDS) for 30 minutes at 55°C and re‐probed with antibodies to each protein corresponding to the first antibodies. Bands detected by ECL chemiluminescence were scanned, and chemiluminescence signal intensities were quantified using the ImageJ program (US National Institutes of Health; http://rsb.info.nih.gov/ij/). The level of phosphorylation or amount of each protein was determined as the ratio of the band intensity of first blots to that of the second blots.

### 2.7 Flow cytometry

Differentiation was assessed by flow cytometry assays of CD11b expression on cell surfaces. HL‐60 and DHL‐60 cells (5 × 10^5^ cells each) were incubated with PE‐conjugated anti‐human CD11b or PE‐conjugated mouse IgG1k isotype for 30 minutes on ice. The cells were washed twice with PBS, resuspended in PBS, and analyzed by flow cytometry.

### 2.8 Kras pull‐down assays

Kras activity was assayed using Kras Activation Assay Kits (Cell Biolabs, Inc) according to the manufacturer's protocol. Briefly, HL‐60 and DHL‐60 cells (2 × 10^7^ cells each) were lysed in 1 ml lysis buffer (25 mmol/L HEPES, pH 7.5, 150 mmol/L NaCl, 1% NP‐40, 10 mmol/L MgCl_2_, 1 mmol/L EDTA, 2% glycerol), and the lysates were incubated with Raf1 RBD agarose beads for 3 hours. at 4°C. The beads were collected by centrifugation and washed thrice with assay buffer. Kras was eluted from beads using Laemmli sample buffer. The samples were subjected to SDS‐PAGE, and pull‐down of active Kras was assessed by Western blotting with anti‐Kras antibody. The blots were scanned and quantified using ImageJ software (NIH).

### 2.9 Lef/Tcf‐sensitive transcriptional reporter gene assay

HL‐60 or DHL‐60 cells were transfected with either Super8x TOPFlash (M50) or Super8x FOPFlash (M51). After 24 hours, the cells were harvested and lysed in a reporter gene lysis buffer. Luciferase activity of the cell extracts was assayed according to the manufacturer's instructions (Promega Corp, Madison, WI) and normalized relative to the protein concentration of the respective lysate. In separate experiments, cells were co‐transfected with siRNAs and M50. Cells were cultured in medium with 1.3% DMSO for 4 days. Cell lysates were assayed for luciferase activity.

### 2.10 Statistical analysis

Data are presented as the mean ± SE. Statistical analysis was performed by Student's *t* tests.

## 3 RESULTS

### 3.1 Kras translocates to the plasma membrane and is activated during DMSO‐induced differentiation of HL‐60 cells

**Figure 1:**
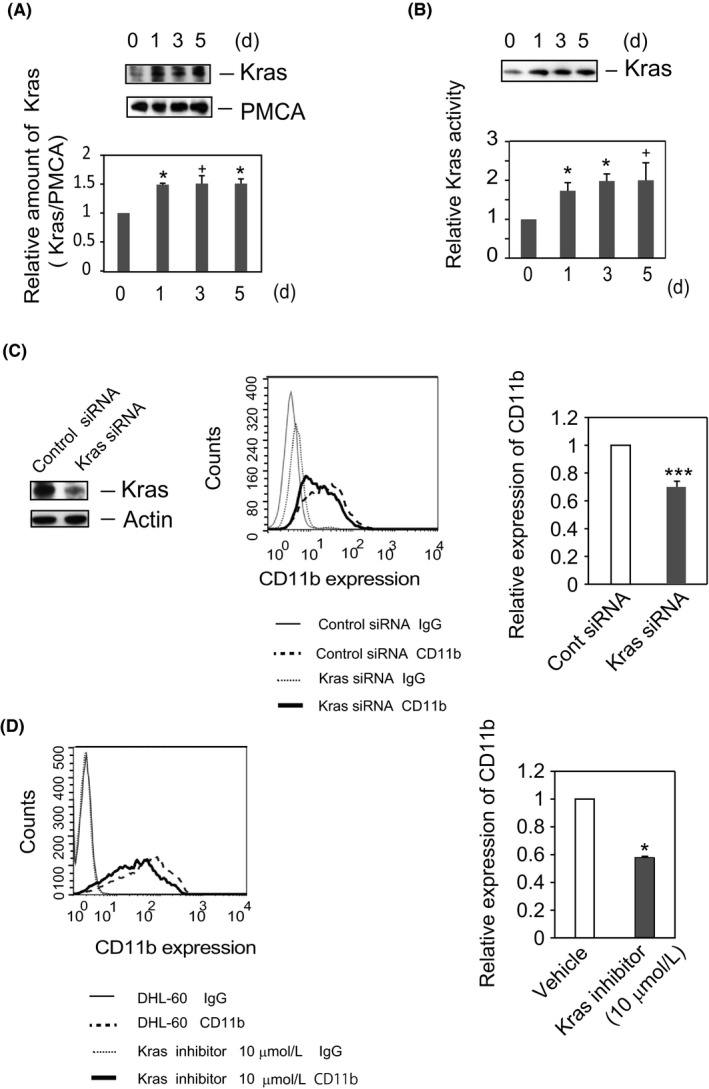
Kras positively regulates the DMSO‐induced differentiation of HL‐60 cells. (A) *The amount of Kras is increased in plasma membranes during differentiation of HL‐60 cells*. HL‐60 cells were cultured in RPMI medium, with or without 1.3% DMSO, for the indicated times. Plasma membrane fractions were prepared, subjected to SDS‐PAGE, and analyzed by Western blotting with antibody to Kras, followed by incubation with anti‐PMCA antibody, as described in *the Materials and Methods*. The blots shown are representative of five independent experiments (upper panel). Quantified Kras levels (Kras/PMCA) are displayed as ratio to those in HL‐60 cells (time = 0) and shown as the means ± SE of five independent experiments. *P < 0.05, ^+^ *P* < 0.1. (B) *Kras is activated in DHL‐60 cells*. Kras activity was assayed using Kras Activation Assay Kits. HL‐60 or DHL‐60 cell lysates were incubated with Raf1 RBD agarose to capture the active form of Kras. The amounts of bound proteins were analyzed by Western blotting with anti‐Kras antibody. A representative blot of four independent experiments is shown (upper panel). Active Kras was detected by immunoblotting with anti‐Kras antibody. The amounts of the active form of Kras were calculated relative to the numbers of cells, and the time course of the ratio of the normalized amount of activated Kras relative to that of resting cells (HL‐60 cells at *t* = 0) was determined. Quantified results are shown as means ± SE of four independent experiments. *P < 0.05, ^+^ *P* < 0.1. (C) *Knockdown of Kras attenuates CD11b expression in DHL‐60 cells*. HL‐60 cells were transfected with the indicated siRNA and cultured in RPMI medium containing 1.3% DMSO for 4 days. Cell lysates were analyzed by Western blotting with anti‐Kras or anti‐actin antibody. The blot shown is representative of eight independent experiments (left panel). CD11b expression was analyzed by flow cytometry. Data representative of four independent experiments are shown (middle panel). IgG; normal IgG‐staining cells. The ratio relative to the cells transfected with control siRNA is shown as mean ± SE of four independent experiments (right panel). ***P < 0.005. (D) *Kras inhibitor attenuates the expression of CD11b in DHL‐60 cells*. Cells were cultured in RPMI medium containing DMSO (1.3%) with or without Kras inhibitor (10 μmol/L) for 5 days. CD11b expression was analyzed by flow cytometry. Data representative of four independent experiments are shown (left panel). The ratio of the geometric mean of CD11b expression on the cells treated with Kras inhibitor to that on cells treated with vehicle is shown as the mean ± SE of four independent experiments (right panel). *P < 0.05

N‐ and Kras mutations have been observed in some patients with AML, although mutations in *ras* oncogenes alone are not sufficient to induce leukemogenesis. The precise roles of *ras* oncogenes in AML remain unclear. Knockout studies have suggested that Kras has specific functions in signaling pathways not shared by other members of *ras* family.^4^ We therefore assessed whether wild‐type Kras has distinct and novel functions in the differentiation of HL‐60 cells. Wild‐type Kras is present in the HL‐60 cells used in this study (data not shown). Ras activity relies on proper anchoring to the plasma membrane, and Ras proteins activate signaling pathways by recruiting effectors to the membrane. We therefore assessed whether the amounts of Kras in the plasma membrane change during DMSO‐induced differentiation of HL‐60 cells. Immunoblotting of DHL‐60 cells with anti‐Kras antibody showed that the amounts of Kras in plasma membrane fractions were increased during differentiation of HL‐60 cells, whereas Western immunoblotting with antibody to PMCA, a plasma membrane marker protein, showed that the amounts of PMCA were unchanged during differentiation (Figure 1A). The total amount of Kras in these cells, however, was unchanged during differentiation (Figure S1), indicating that Kras is translocated to the plasma membrane during DMSO‐induced differentiation of HL‐60 cells.

Although Kras was not fully active in undifferentiated HL‐60 cells, DMSO‐induced differentiation increased the Kras activity in whole cell lysates by 1.75‐fold, an increase sustained for 5 days after treatment with DMSO (Figure 1B). This finding that Kras was activated upon DMSO treatment suggested that Kras activation is involved in the DMSO‐induced differentiation of HL‐60 cells.

### 3.2 Kras knockdown or treatment with Kras inhibitor attenuates CD11b expression in DHL‐60 cells

To further investigate the roles of Kras in HL‐60 cell differentiation, these cells were treated with Kras or control siRNA for 4 days in the presence of DMSO. Kras siRNA reduced the level of Kras protein to about 35% of that in control siRNA–treated cells (Figure 1C) and the expression of CD11b protein to 68% of that in control siRNA–treated cells, indicating that Kras is involved in the DMSO‐induced differentiation of HL‐60 cells. In the absence of DMSO, however, the expression of CD11b was unaffected by transfection with either control or Kras siRNA (Figure S2).

To further assess the functions of Kras in DHL‐60 cells, HL‐60 cells were cultured with DMSO in the presence of the Kras inhibitor SAH‐SOS1A or vehicle for 5 days. Similar to the transfection of Kras siRNA, Kras inhibitor attenuated the expression of CD11b by 40% compared with vehicle (Figure 1D). Taken together, these findings demonstrate that Kras mediates the DMSO‐induced differentiation of HL‐60 cells.

### 3.3 Inhibition of GSK3β enhances DMSO‐induced differentiation of HL‐60 cells

**Figure 2:**
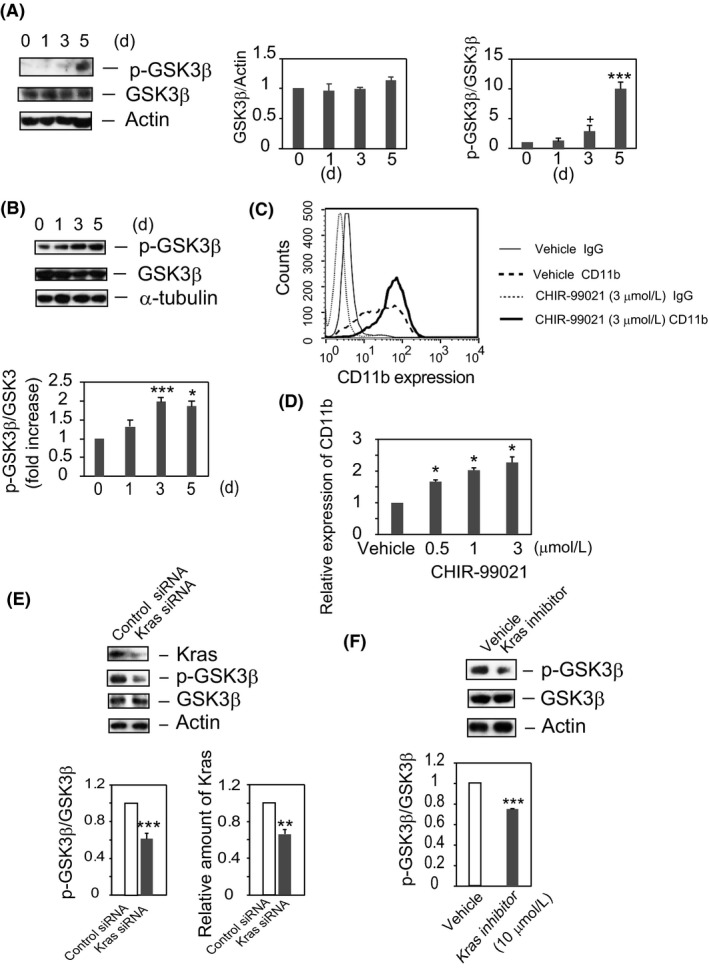
GSK3β is phosphorylated and Kras acts upstream of GSK3β in DHL‐60 cells. (A) *GSK3β is markedly phosphorylated in DHL‐60 cells*. Lysates were analyzed by SDS‐PAGE and Western blotting with the indicated antibodies. The blots shown are representative of four independent experiments (left panel). The time courses of the GSK3β/actin ratio (middle panel) and of the phosphorylation ratios (p‐GSK3β/GSK3β) relative to resting cells (HL‐60 cells at *t* = 0) (right panel) are shown as the means ± SE of four independent experiments. ^+^ *P* < 0.1, ***P < 0.005. (B) *DMSO enhances GSK3β phosphorylation in cytoplasm*. Cytoplasm fractions were analyzed by Western blotting with anti‐p‐GSK3β, anti‐GSK3β, and anti‐α‐tubulin antibodies. The blots shown are representative of four independent experiments (upper panel). The time courses of the phosphorylation ratios (p‐GSK3β/GSK3β) relative to resting cells (HL‐60 cells at *t* = 0) are shown as the means ± SE of four independent experiments (lower panel). *P < 0.05, ***P < 0.005. (C) *Inhibition of GSK3β enhances the expression of CD11b in DHL‐60 cells*. HL‐60 cells were cultured in RPMI medium containing 1.3% DMSO plus CHIR‐99021 (3 μmol/L) or vehicle for 5 days. Expression of CD11b was determined by flow cytometry. The results shown are representative of three independent experiments. (D) *Expression of CD11b is dose‐dependently enhanced by CHIR‐99021*. The ratios of CD11b expression (geometric mean) of cell treated with CHIR‐99021 (0.5, 1, 3 μmol/L) relative to solvent control–treated cells (vehicle) are shown as the means ± SE of three independent experiments. *P < 0.05. (E) *Knockdown of Kras inhibits phospho‐GSK3β generation in DHL‐60 cells*. Cells transfected with Kras or control siRNA were cultured in RPMI medium plus 1.3% DMSO for 4 days. Cell lysates were analyzed by Western blotting with anti‐Kras, anti‐p‐GSK3β, anti‐GSK3β, or anti‐actin antibody. The results shown are representative of 10 independent experiments (upper panel). The bands were quantified, with phosphorylation ratios (p‐GSK3β/GSK3β, lower left panel, ***P < 0.005) and Kras levels (lower right panel, **P < 0.01) relative to cells transfected with control siRNA are shown as the means ± SE of 10 independent experiments. (F) *Kras inhibitor inhibits GSK3β phosphorylation*. Cells were cultured in RPMI medium or RPMI medium plus 1.3% DMSO with or without Kras inhibitor (SAH‐SOS1A). Cell lysates were analyzed by immunoblotting with antibodies to p‐GSK3β, GSK3β, and actin. The results shown are representative of eight independent experiments (upper panel). The bands were quantified, with the result shown being the mean ± SE of eight independent experiments. ***P < 0.005

The serine‐/threonine‐specific protein kinase GSK3β plays a pivotal role in the Wnt canonical pathway.^32^ Phosphorylation of GSK3β is a good indicator of the Wnt canonical pathway, as phosphorylation of GSK3β at Ser 9 inhibits GSK3β activity.^33^ In the absence of Wnt signaling, β‐catenin is degraded by a complex of proteins, including GSK3β, APC, CK1, protein phosphatase 2A, and E3‐ubiquitin ligase β‐TrCP. Phosphorylation of β‐catenin by GSK3β and CK1 regulates its stability.^18^, ^19^, ^22^ To investigate the possibility of cross talk between the Wnt and Kras signaling pathways during the differentiation of HL‐60 cells, we first assessed whether DMSO treatment induces GSK3β phosphorylation (Figure 2A). Phosphorylation of GSK3β (Ser 9), which was almost undetectable in resting HL‐60 cells, was enhanced 10‐fold by treatment with DMSO for 5 days, whereas total amounts of GSK3β were unchanged (Figure 2A), indicating that the Wnt/β‐catenin pathway participates in the DMSO‐induced differentiation of HL‐60 cells. To more precisely analyze these findings, we investigated the level of phosphorylated GSK3β in cytoplasm. Although the amount of GSK3β in cytoplasm was unaffected by DMSO, GSK3β phosphorylation (p‐GSK3β/GSK3β) was enhanced twofold after treatment with DMSO for 3 days and was sustained for up to 5 days (Figure 2B).

Because the GSK3β inhibitor CHIR‐99021 has been found to activate Wnt signaling,^34^ the involvement of GSK3β in the DMSO‐induced differentiation of HL‐60 was assessed by treating these cells with CHIR‐99021. HL‐60 cells were incubated with DMSO in the presence or absence of CHIR‐99021 (3 μmol/L) for 5 days. Flow cytometry analysis showed that CHIR‐99021 dose‐dependently enhanced CD11b expression (Figure 2C, 2D), indicating that the suppression of GSK3β activity is an important step in the DMSO‐induced differentiation of HL‐60 cells and suggesting the involvement of the Wnt/β‐catenin pathway.

### 3.4 Phosphorylation of GSK3β is attenuated by inhibition of Kras

To determine whether GSK3β acts downstream of Kras, the phosphorylation status of GSK3β was assessed in cells transfected with Kras siRNA. HL‐60 cells were transfected with Kras or control siRNA, and differentiation was induced by DMSO. Compared with cells treated with control siRNA, cells treated with Kras siRNA showed a 35% reduction in Kras protein level (Figure 2E, right panel). Kras siRNA also reduced the phosphorylation of GSK3β at Ser 9 by 40% compared with control siRNA, although the total amounts of GSK3β in these cells were unchanged (Figure 2E, left panel). To analyze the effects of a Kras inhibitor on GSK3β phosphorylation, cells were treated with Kras inhibitor or vehicle in the presence of DMSO for 5 days, and GSK3β phosphorylation status was determined. Although similar amounts of GSK3β and actin were present in lysates, GSK3β phosphorylation (p‐GSK3β/GSK3β) was 35% lower in cells treated with Kras inhibitor than with vehicle (Figure 2F), indicating that GSK3β is a downstream effector of Kras.

### 3.5 PI3K/AKT is an effector molecule for Kras signaling during DMSO‐induced differentiation of HL‐60 cells

**Figure 3:**
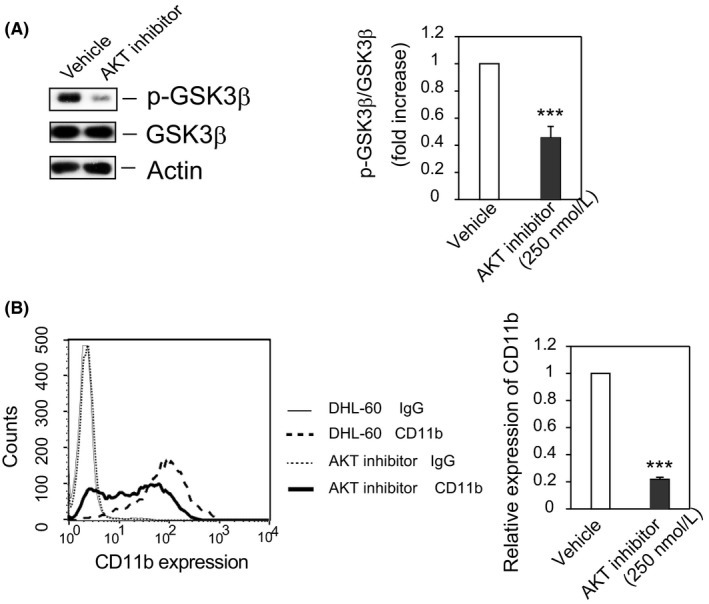
AKT acts upstream of GSK3β in DHL‐60 cells. (A) *AKT is responsible for the phosphorylation of GSK3β in DHL‐60 cells*. Cells were cultured in RPMI medium plus 1.3% DMSO with AKT inhibitor (AKTi‐1/2, 250 nmol/L) or vehicle for 5 days, and cell lysates were analyzed by Western blotting with the indicated antibodies. The results shown are representative of four independent experiments (left panel). Bands were quantified and the ratios of phosphorylated to total GSK3β shown as the mean ± SE of four independent experiments (right panel). ***P < 0.005. (B) *AKT inhibitor attenuates the expression of CD11b in DHL‐60 cells*. CD11b expression was assayed by flow cytometry, with the results shown being representative of three independent experiments (left panel). The ratio of CD11b expression (geometric mean) by AKT inhibitor–treated cells to that by DMSO‐treated cells (vehicle) is shown as the mean ± SE of three independent experiments (right panel). ***P < 0.005

Because PI3K/AKT plays a crucial role in Kras signaling during hematopoiesis,^35^ and because GSK3β has been identified as an AKT (protein kinase B) substrate,^36^ we assessed whether the PI3K/AKT pathway may be linked to Kras and the differentiation of HL‐60 cells. To demonstrate a direct link between AKT and GSK3β, HL‐60 cells were incubated with the AKT inhibitor AKTi‐1/2 or vehicle for 5 days in the presence of DMSO. The p‐GSK3β/GSK3β ratio was about 50% lower in cells treated with AKTi‐1/2, suggesting that AKT is the kinase responsible for phosphorylation of GSK3β (Figure 3A). Furthermore, CD11b expression was reduced to 20% compared with vehicle‐treated cells (Figure 3B). PI3K inhibitor also suppressed both GSK3β phosphorylation and CD11b expression, but did not alter GSK3β expression (Figure S3A, B). These findings confirmed that PI3K/AKT is a downstream effector of Kras signaling and that AKT phosphorylates GSK3β.^35^, ^37^

### 3.6 β‐catenin and Tcf4 accumulate in the nuclear fraction of DHL‐60 cells

**Figure 4:**
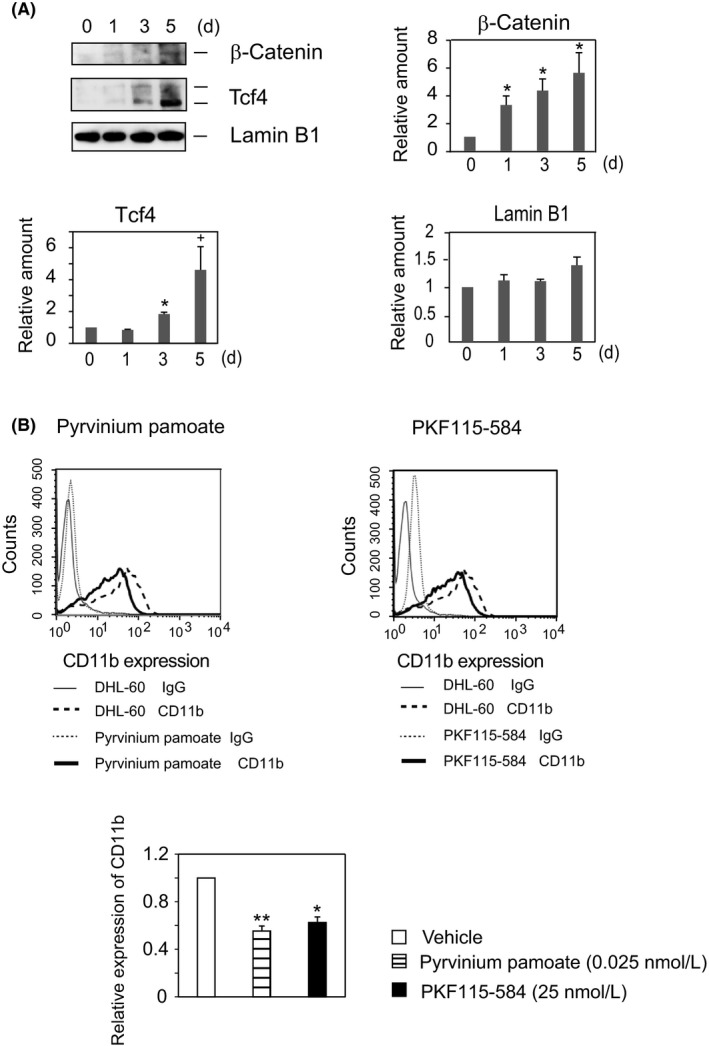
Accumulation of β‐catenin in nuclei regulates DMSO‐induced differentiation of HL‐60 cells. (A) *Accumulation of β‐catenin and Tcf4 in the nuclear fraction in response to DMSO*. Nuclear fractions prepared using Nuclear Extract Kits (Active Motif) were analyzed by Western blotting with anti‐β‐catenin, anti‐Tcf4, and anti‐Lamin B1 antibodies. The results shown are representative of six independent experiments (upper left panel). Time course of the expression of β‐catenin (upper right panel), Tcf4 (lower left panel), and Lamin B1 (lower right panel) in differentiated cells relative to resting cells (HL‐60 cells at *t* = 0). The results are expressed as the means ± SE of six independent experiments. ^+^ *P* < 0.1, *P < 0.05. (B)* β‐catenin inhibitors attenuate the expression of CD11b in DHL‐60 cells*. HL‐60 cells were cultured for 5 days with pyrvinium pamoate (0.025 nmol/L) or RKF115‐584 (25 nmol/L) in the presence of DMSO (1.3%), and CD11b expression was determined by flow cytometry. Each of the flowcharts is representative of three independent experiments (upper panels). Ratios of CD11b expression (geometric mean) of the inhibitor‐treated cells relative to solvent control (vehicle), expressed as the means ± SE of three independent experiments (lower panel). *P < 0.05, **P < 0.01

Inhibition of GSK3β‐catalyzed phosphorylation of β‐catenin requires a dynamic multiprotein complex, which includes APC, Axin, and Dishevelled.^22^ Because Wnt stimulation triggers shuttling of Axin and β‐catenin to the nucleus,^38^ one of the hallmarks of activated Wnt signaling is the accumulation of β‐catenin in the nucleus.^21^, ^22^, ^23^We found that inhibition of GSK3β upregulated CD11b expression in DHL‐60 cells. To confirm the involvement of Wnt/β‐catenin signaling in the DMSO‐induced differentiation of HL‐60 cells, we assessed the expression of β‐catenin and its transcription factor Tcf4 in cell lysates. In agreement with previous findings,^39^ we observed slight expression of β‐catenin in resting and early DHL‐60 cells, with higher levels of β‐catenin and Tcf4 observed only after DMSO treatment for 5 days (Figure S4). The elevated levels of p‐GSK3β, β‐catenin, and Tcf4 in cell lysates suggested that β‐catenin and Tcf4 may accumulate in the nucleus during the differentiation of HL‐60 cells. We found that β‐catenin started to accumulate significantly after 1 day of treatment with DMSO, reaching its maximum level after incubation for 5 days with DMSO (Figure 4A). In contrast, Tcf4 accumulation in the nucleus was detected only after incubation with DMSO for 3 days. Obvious accumulation of Tcf4 followed the elevation of β‐catenin in the nucleus, whereas levels of Lamin B1, a marker protein of the nuclear fraction, were similar in DMSO‐differentiated and resting HL‐60 cells. Thus, the amounts of p‐GSK3β, β‐catenin, and Tcf4 correlated with each other, indicating that the Wnt/β‐catenin pathway is involved in the differentiation of HL‐60 cells. Increased levels of p‐GSK3β stabilize β‐catenin, which, in turn, acts as a nuclear transcriptional co‐activator with the Lef/Tcf family to stimulate the transcription of various target genes in DHL‐60 cells.

### 3.7 Treatment with β‐catenin inhibitors attenuates CD11b expression in DHL‐60 cells

As reported previously,^39^ resting and early differentiated HL‐60 cells contain low levels of β‐catenin, with the accumulation of β‐catenin in the nucleus correlating with the degree of differentiation of HL‐60 cells (Figure S4 and Figure 4A). In addition, β‐catenin expression is upregulated in most patients with AML.^20^ Because the functions of β‐catenin have not yet been determined, we assessed whether β‐catenin accumulation in the nucleus promotes the DMSO‐induced differentiation of HL‐60 cells. Two unrelated compounds, pyrvinium pamoate and PKF115‐584, have been found to inhibit the Wnt signaling pathway, pyrvinium pamoate by blocking the transcription of the β‐catenin gene and PKF115‐584 by being a potent and specific small‐molecule inhibitor of β‐catenin/Tcf4 interactions.^40^ Because both compounds attenuate Tcf4 transcriptional activity, we tested their effects on DMSO‐induced differentiation of HL‐60 cells. At concentrations lower than their IC50s, pyrvinium pamoate (10 nmol/L) and PKF115‐584 (0.4 μmol/L) attenuated the expression of CD11b by about 40% (Figure 4B). Treatment with PKF115‐584 had no effect on cell growth, whereas pyrvinium pamoate enhanced DHL‐60 cell proliferation ~10%. Both inhibitors significantly attenuated CD11b expression, indicating that β‐catenin enhances DMSO‐induced differentiation of HL‐60 cells.

### 3.8 DMSO activates Lef/Tcf‐sensitive transcription

**Figure 5:**
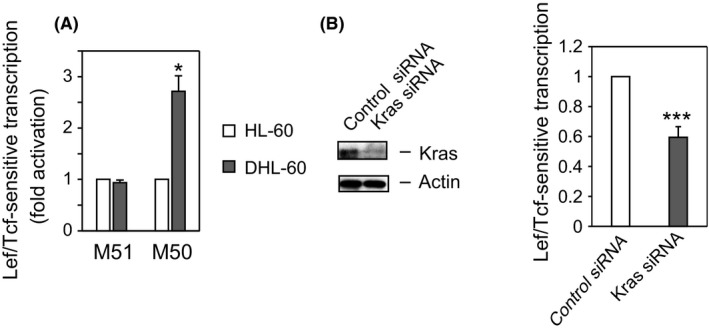
DMSO promotes activation of Lef/Tcf‐sensitive transcription. (A) *DMSO enhances Lef/Tcf‐sensitive transcription*. HL‐60 cells were cultured in RPMI medium without (HL‐60) or with (DHL‐60) 1.3% DMSO for 4 days, followed by co‐transfection for one day with Super8x TOPFlash (M50) or Super8x FOPFlash (M51). The ratio of the activation of Lcf/Tcf‐sensitive transcription in DHL‐60 cells to that in HL‐60 cells is shown as the mean ± SE of four independent experiments. *P < 0.05. (B) *Knockdown of Kras attenuates Lef/Tcf‐sensitive transcription in response to DMSO*. HL‐60 cells were co‐transfected with the indicated siRNA and M50, and cultured in medium containing 1.3% DMSO for 4 days. Cell lysates were analyzed by Western blotting with anti‐Kras and anti‐actin antibodies (left panel) and for Lef/Tcf‐sensitive transcription (right panel). The ratio of the activation of Lcf/Tcf‐sensitive transcription in Kras siRNA–treated DHL‐60 cells to that in control siRNA–treated DHL‐60 cells is shown as the mean ± SE of six independent experiments. ****P < 0.005

Because activation of Lef/Tcf‐sensitive transcription by β‐catenin is an early outcome in the Wnt canonical pathway,^18^ we analyzed the activity of a Lef/Tcf‐sensitive reporter gene, the M50 construct, which harbors multiple β‐catenin binding sites in its promoter. The DMSO‐induced enhancement of Lef/Tcf transcription activity was found to correlate with the accumulation of β‐catenin in the nucleus, and stimulation with DMSO for 4 days triggers M50 reporter genes (Figure 5A). In contrast, DMSO had no effect on the activity of the control gene M51, which lacks β‐catenin binding sites in its promoter. To confirm the involvement of the Kras/Wnt/β‐catenin signaling network in the DMSO‐induced differentiation of HL‐60 cells, HL‐60 cells were transfected with control or Kras siRNA, along with Super TOPFlash (M50), and cultured in medium containing 1.3% DMSO for 4 days. Lef/Tcf‐sensitive transcription was 40% lower in cells transfected with Kras siRNA (Figure 5B), suggesting that the Wnt/β‐catenin signaling is downstream of Kras.

### 3.9 DMSO enhances C/EBPα and C/EBPɛ expression in a time‐dependent manner

**Figure 6:**
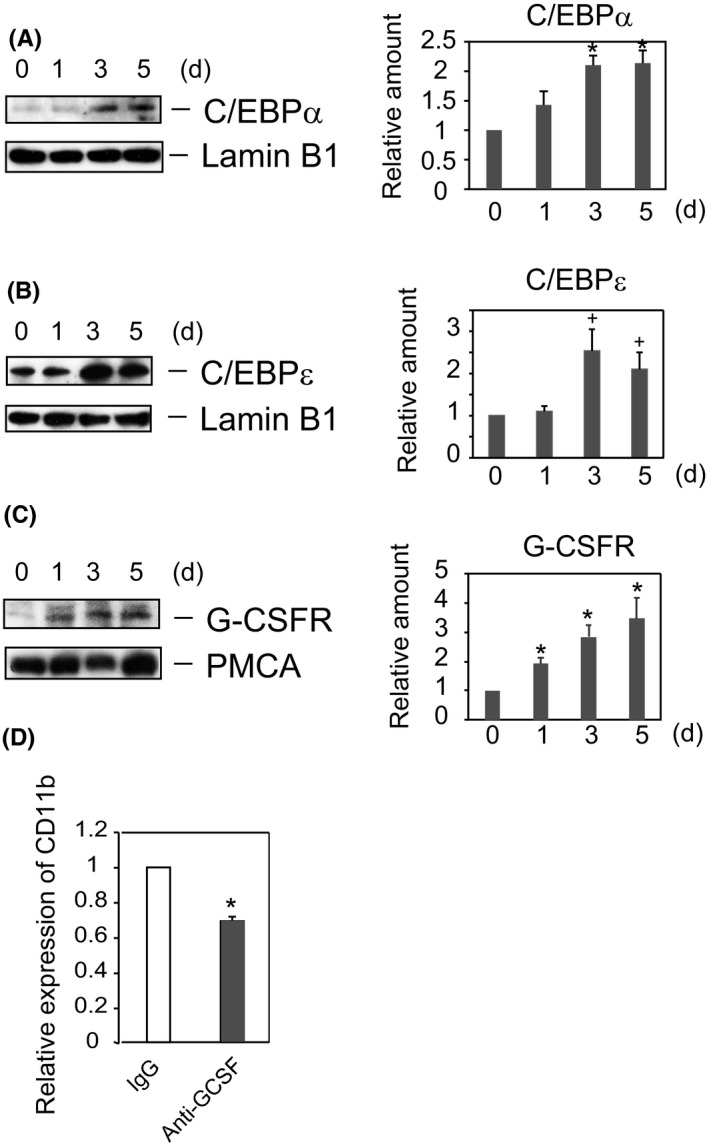
C/EBPα, C/EBPɛ, and G‐CSF receptor are upregulated in HL‐60 cells by DMSO treatment. (A, B, C) *Expression of C/EBPs and G‐CSF receptor was enhanced in DHL‐60 cells*. HL‐60 cells were cultured in RPMI medium with or without 1.3% DMSO for the indicated times. Nuclear fractions were prepared using Nuclear Extract Kits (Active Motif) (A, B). In separate experiments, plasma membrane fractions were prepared (C). Protein expression was analyzed by Western blotting with antibodies to C/EBPα (A), C/EBPɛ (B), Lamin B1 (A, B), G‐CSFR (C), and PMCA (C). Results of blotting are representative of four independent experiments. The ratios of protein expressions in DHL‐60 relative to HL‐60 cells at *t* = 0 (right panels), expressed as the mean ± SE of four independent experiments. *P < 0.05, ^+^ *P* < 0.06. (D) *Anti‐G‐CSF antibody attenuates the DMSO‐induced differentiation of HL‐60 cells*. HL‐60 cells were cultured in RPMI medium with 1.3% DMSO in the presence of either anti‐G‐CSF or normal IgG for 5 days, and the expression of CD11b was determined by flow cytometry. The ratios of CD11b expression (geometric mean) of anti‐G‐CSF IgG‐treated cells relative to normal IgG‐treated cells are shown as the mean ± SE of three independent experiments. *P < 0.05

We subsequently assessed the transcription factors that were upregulated following β‐catenin accumulation in the nucleus during DMSO‐induced differentiation of HL‐60 cells. CCAAT/enhancer‐binding proteins (C/EBPs) are transcription factors involved in regulating cell differentiation and proliferation.^41^ C/EBPα and C/EBPε regulate neutrophil maturation,^42^ Lef‐1 regulates the expression of C/EBPα during granulopoiesis 27 and the expression of C/EBPε peak in mature neutrophils and macrophages.^29^, ^42^, ^43^ We therefore assessed whether C/EBPα and C/EBPε were upregulated during DMSO‐induced differentiation of HL‐60 cells. C/EBPα started to accumulate after 1 day of treatment with DMSO, peaking after 3 ~ 5 days (Figure 6A). In contrast, DMSO had no effect on C/EBPε accumulation after 1 day, but enhanced its accumulation after 3 days (Figure 6B), with enhancement of C/EBPε expression occurring following upregulation of C/EBPα. Overlapping expression of C/EBPα and C/EBPε was observed after 3 days of treatment with DMSO. These time lags in the expression of C/EBPα and C/EBPε have been observed previously.^29^, ^42^

Another critical regulator of granulopoiesis is granulocyte colony‐stimulating factor (G‐CSF), a cytokine that regulates the differentiation and proliferation of myeloid cells.^44^ G‐CSF receptor has binding sites for C/EBPα and PU.1, regulating several myeloid gene promoters.^45^ Increased G‐CSF receptor protein expression was observed after 1 day of treatment with DMSO, with G‐CSF receptor expression increasing in a time‐dependent manner (Figure 6C). In contrast, DMSO had little effect on PMCA levels in plasma membranes. To confirm the significance of G‐CSF receptor in the differentiation of HL‐60 cells, we investigated the effect of anti‐G‐CSF antibody on DMSO‐induced differentiation of HL‐60 cells, finding that treatment with anti‐G‐CSF antibody inhibited CD11b expression (Figure 6D).

## 4 DISCUSSION

The Ras family of small GTPases consists of three isoforms: Hras, Nras, and Kras. Activated Ras proteins mediate distinct biological processes, including cell proliferation, differentiation, and apoptosis, by recruiting downstream effectors. In many human cancers, Kras is the most frequently mutated protein. The Wnt/β‐catenin pathway has been implicated in many cellular signaling events.^17^, ^18^, ^19^, ^22^ This pathway is well controlled to maintain normal hematopoiesis,^46^ with β‐catenin playing pivotal roles in controlling the balance between cell proliferation and differentiation.^20^ Moreover, β‐catenin is a central player in the canonical cascade, with its stability regulated by a destruction complex. Inactivation of GSK3β by phosphorylation (Ser 9) triggers the release of β‐catenin from the destruction complex.^47^ Constitutive activation of Wnt signaling results in malignancy and blocks differentiation. Cross talk between oncogenic Kras and Wnt/β‐catenin signaling has been reported in colorectal cancers, with its activity mediated through GSK3β or LRP6.^48^, ^49^ Kras^Val12^ enhanced the level of β‐catenin by inhibiting GSK3β, although this effect was not mediated by phosphorylation of GSK3β at Ser 9.^49^ The expression of p21Ras proteins is upregulated in the differentiation of HL‐60 cells,^14^ but the involvement of Kras in myeloid differentiation is less clear. The current study focused on the properties of Kras other than its oncogenic activity. Although the functional significance of wild‐type Kras protein has not yet been clearly established, wild‐type *Kras* is regarded as a tumor suppressor, which is frequently lost during the progression of many cancers.^11^ The finding that wild‐type Kras inhibits oncogenic Kras‐induced T‐cell leukemia suggests that Kras can also act as a tumor suppressor,^12^ and loss of wild‐type Kras was found to promote the activation of all Ras isoforms in oncogenic Kras‐induced leukemogenesis.^13^ Similarly, our results demonstrate that the Kras signaling pathway acts as tumor suppressor during DMSO‐induced differentiation of HL‐60 cells.

Neutrophilic granulocytes have a short life, indicating that granulopoiesis should be tightly regulated. The C/EBPs are a family of transcription factors that are critical for normal cell differentiation, with C/EBPs being important regulators of cytokine expression in neutrophils.^50^ Neutrophilic differentiation depends on C/EBPα, and Lef‐1 regulates the expression of C/EBPα during granulopoiesis.^27^ C/EBPα upregulates the G‐CSF receptor promoter in myeloid cells,^45^, ^51^ whereas C/EBPɛ is a critical regulator of terminal granulopoiesis.^42^ C/EBPɛ regulation is a rate‐limiting step during G‐CSF‐regulated granulocyte differentiation.^44^ Treatment of HL‐60 cells with DMSO for 3 days resulted in the upregulation of both the C/EBPδ and C/EBPɛ genes.^30^

**Figure 7:**
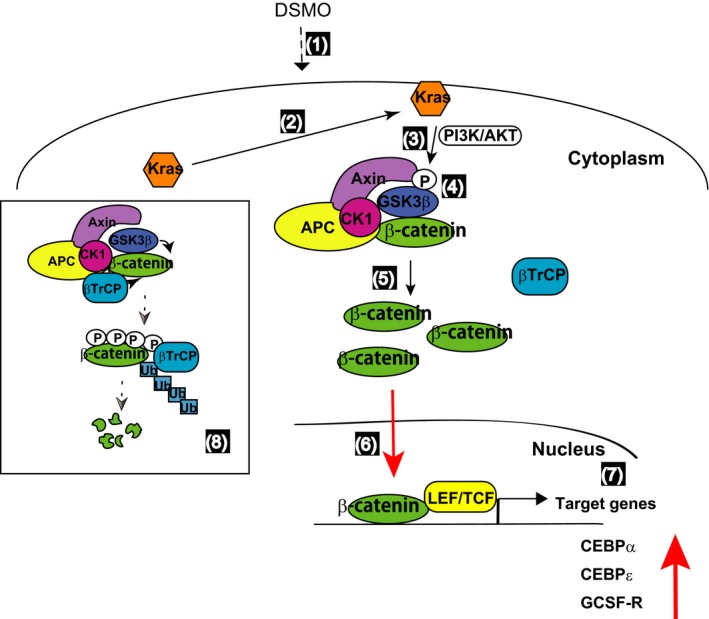
Novel Kras‐Wnt/β‐catenin signaling networks in the DMSO‐induced differentiation of HL‐60 cells. DMSO triggers Kras activation through the recruitment of Kras to the plasma membrane (1, 2). AKT phosphorylates GSK3β and inactivates GSK3β (3, 4). Accumulated unphosphorylated β‐catenin in the cytoplasm translocates to the nucleus (5, 6), where it binds to Lef/Tcf and stimulates the transcription of target genes such as C/EBPα, C/EBPɛ, and G‐CSF receptor (7). In the absence of DMSO, β‐catenin is phosphorylated by a destruction complex composed of the core proteins, including Axin, APC, CK1, β‐catenin, GSK3β, and E3‐ubiquitin ligase β‐TrCP. Degradation of phosphorylated β‐catenin follows its ubiquitination by proteasomes (8)

Despite DMSO‐induced differentiation of HL‐60 cells being well‐known, the mechanism by which DMSO triggers the differentiation of HL‐60 cells remains unclear. In assessing this mechanism, we found, for the first time, that the Kras‐PI3K/AKT‐Wnt/β‐catenin networks were novel key signaling elements in DMSO‐induced HL‐60 cell differentiation. Proposed pathways are schematically described in Figure 7. We found that wild‐type Kras positively regulated the DMSO‐induced differentiation of HL‐60 cells and that there was cross talk between Kras and the Wnt/β‐catenin network during differentiation. The expression of CD11b, a marker for granulocytic differentiation, was attenuated by either Kras knockdown or a Kras inhibitor, as well as by an AKT inhibitor and small‐molecule inhibitors of β‐catenin. In contrast, CD11b expression was enhanced by GSK3β inhibitor. Phosphorylation of GSK3β by AKT inactivated GSK3β (Figure 7, (3,4)) and triggered the stabilization of non‐phosphorylated β‐catenin (Figure 7, (5)), whereas β‐catenin and Tcf4 accumulated in the nuclei of DHL‐60 cells (Figure 7, (6)). Accumulation of β‐catenin stimulated Lef/Tcf‐sensitive transcription. β‐Catenin inhibitors blocked CD11b expression, whereas elevated β‐catenin positively regulated the DMSO‐induced differentiation of HL‐60 cells. In the absence of DMSO, phosphorylated β‐catenin is degraded by ubiquitin‐mediated proteolysis (Figure 7, (8)). Indeed, β‐catenin was slightly expressed in undifferentiated HL‐60 cells. Taken together, these findings indicate that β‐catenin plays an important role in HL‐60 cell differentiation.

This study also showed that PI3K/AKT, GSK3β, and β‐catenin were downstream of Kras. GSK3 is a pivotal regulator of hematopoiesis.^52^, ^53^ Although GSK3α deletion alone had no effect on hematopoiesis, deletion of GSK3β resulted in a pre‐neoplastic state consistent with human myelodysplastic syndromes. Furthermore, the knockdown of both GSK3α and GSK3β resulted in aggressive AML.^54^ The GSK3 proteins have been implicated in many signaling pathways and act as both tumor suppressors and tumor enhancers.^33^ Thus, the GSK3s and β‐catenin are important elements in DMSO‐induced differentiation of HL‐60 cells.

The transcription factors C/EBPα and C/EBPɛ are also upregulated and involved in the granulopoiesis of HL‐60 cells. C/EBPα expression, which is necessary to induce the granulocytic differentiation of bipotential cells, was enhanced soon after treatment with DMSO and was sustained until late differentiation. C/EBPɛ, which acts downstream of C/EBPα, was upregulated following the elevation of G‐CSF receptor in DHL‐60 cells. C/EBPα has been shown to upregulate the expression of G‐CSF receptor and C/EBPɛ.^45^, ^51^ Similarly, the present study found an overlap between C/EBPα and C/EBPɛ expression, with C/EBPα expressed first followed by C/EBPɛ.^29^ The importance of C/EBPα in the expression of G‐CSF receptor is supported by results showing that G‐CSF receptor mRNA is selectively absent in C/EBPα knockout mice.^25^ The sustained expression of C/EBPα until the late differentiation of HL‐60 cells induced bipotential myeloid precursors to become granulocytes, not monocytes. C/EBPα mRNA levels were reduced in HL‐60 cells undergoing monocytic differentiation in response to treatment with 12‐O‐tetra‐decanoylphorbol‐13‐acetate (TPA).^26^

The partial inhibition of CD11b expression by treatment with neutralizing anti‐G‐CSF antibody during DMSO‐induced differentiation suggests that DMSO regulates the differentiation of HL‐60 cells by several distinct pathways. Neutrophil development proceeds via both G‐CSF‐dependent and G‐CSF‐independent pathways, as mice lacking both G‐CSF and G‐CSF receptor produce morphologically mature neutrophils, although at lower than normal levels.^55^ Expression of other C/EBPs in these mice can compensate for the lack of a G‐CSF receptor–mediated pathway. C/EBPs are required for granulopoiesis independent of the induction of G‐CSF receptor.^56^ One dispensable activity may be C/EBPα activation of the C/EBPɛ gene,^56^ with C/EBPα replaced by C/EBPβ under stress conditions.^56^, ^57^

In summary, the present study showed that wild‐type Kras regulates the differentiation of HL‐60 cells via the Wnt/β‐catenin signaling pathway. Accumulation of C/EBPα upregulates the expression of G‐CSF receptor and stimulates mature granulopoiesis in HL‐60 cells. Thus, upregulated C/EBPs are necessary for the granulation and maturation of neutrophilic lineage cells upon DMSO treatment. Kras and Wnt‐related molecules are activated during the development of many tumor types, with oncogenic Kras frequently showing enhanced signaling through the Wnt pathway and mediating tumor development. The present study found that activation of Kras and the Wnt/β‐catenin pathway triggered DMSO‐induced differentiation of HL‐60 cells. Canonical Wnt signaling is crucial for T‐cell development and functions in a dose‐dependent fashion.^46^ Complexities of Wnt signaling in hematopoiesis result from Wnt dosage, the source of hematopoietic cells, and interactions with other signaling pathways.^58^ Therefore, finding the optimal kinetics and dose regimes is crucial for prospective translational applications. Fine adjustment of β‐catenin levels governs downstream signaling networks. During DMSO‐induced differentiation of HL‐60 cells, β‐catenin levels are slightly upregulated by Kras signaling networks, not by Wnt protein, and these increases in β‐catenin levels may govern downstream signaling events. Constitutively, high levels of β‐catenin produced by Wnt may result in super proliferation and tumorigenesis. Kras may therefore modulate a fine adjustment of β‐catenin levels during DMSO‐induced differentiation of HL‐60 cells. Indeed, lower fold activation of Lef/Tcf‐sensitive transcription was observed during DMSO‐induced differentiation of HL‐60 cells.

Wild‐type Kras coordinates the DMSO‐induced differentiation of HL‐60 cells through the Wnt canonical pathway. The present study showed the importance of β‐catenin and GSK3β in this process. This finding, in which wild‐type Kras‐PI3K/AKT‐Wnt networks behave in a tumor‐suppressive manner during the differentiation of HL‐60 cells, was a novel result.

## CONFLICT OF INTEREST

The authors declare that they have no conflict of interest.

## AUTHORS CONTRIBUTIONS

NY designed the study. NY and YK performed the experiments and analyzed the data. NY and KI wrote the manuscript. YH, YT, KT, HO, and KI participated in coordination and helped the study. All authors reviewed and approved the manuscript.
