# Supplementary figures and images for: Kras promotes myeloid differentiation through Wnt/β‐catenin signaling
Source: FASEB Bioadv. 2019 Jul 1;1(7):435–49. doi: 10.1096/fba.2019-00004 (PMC6996383; doi:10.1096/fba.2019-00004)

Figure S1 *Yokoyama et al.*

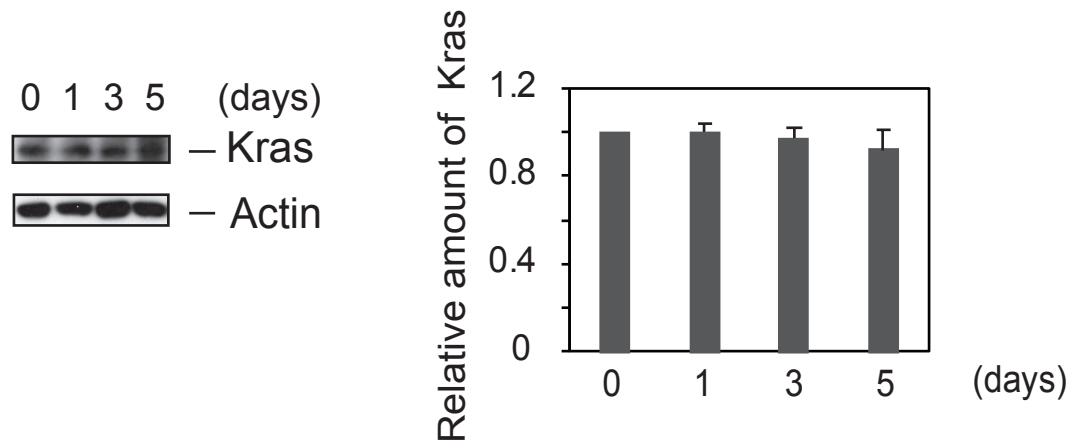

Figure S2 *Yokoyama et al.*

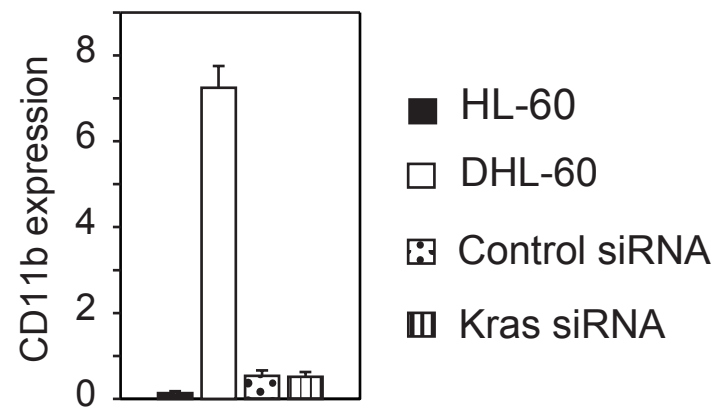

Figure S3 Yokoyama *et al.*

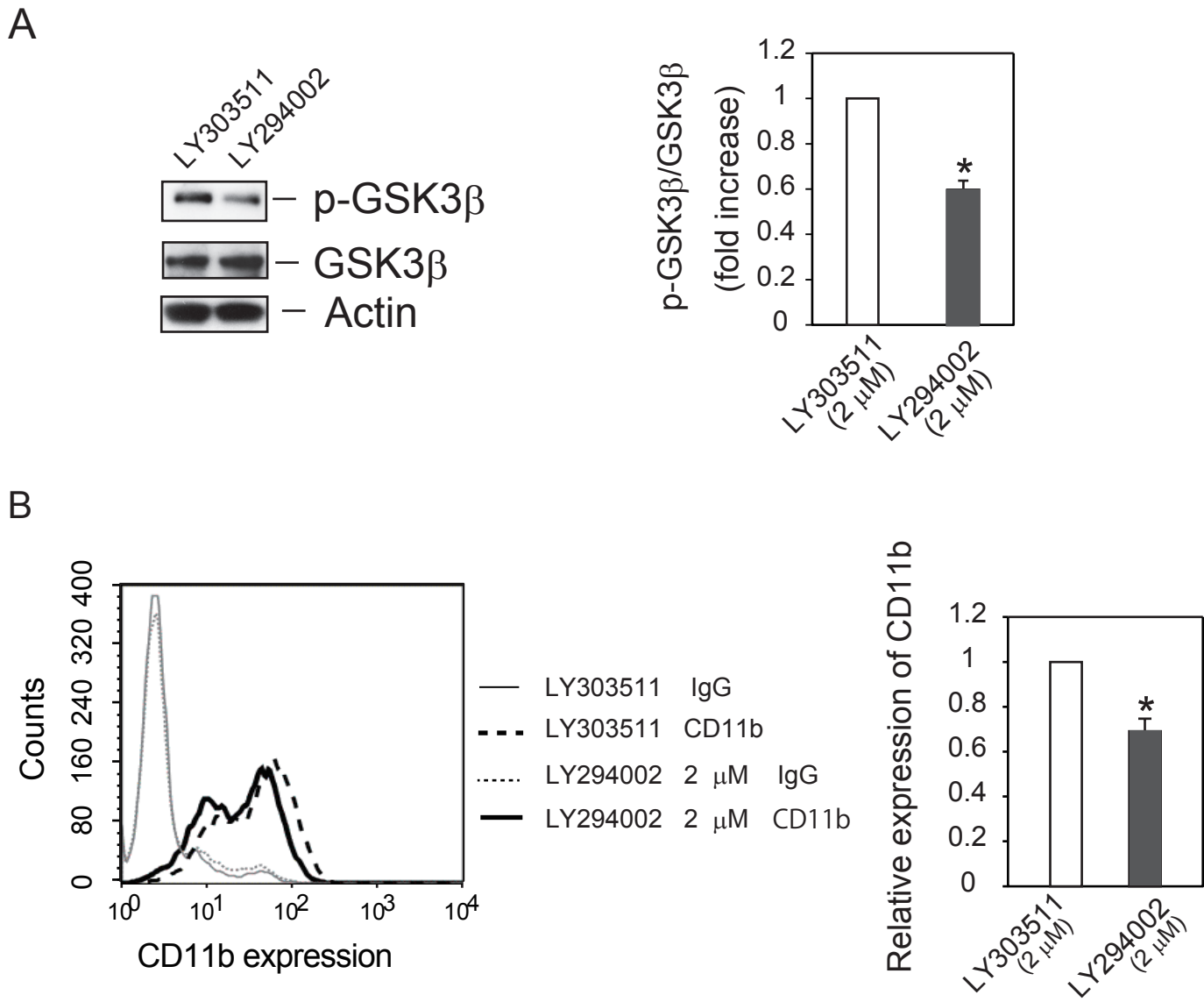

Figure S4 *Yokoyama et al.*

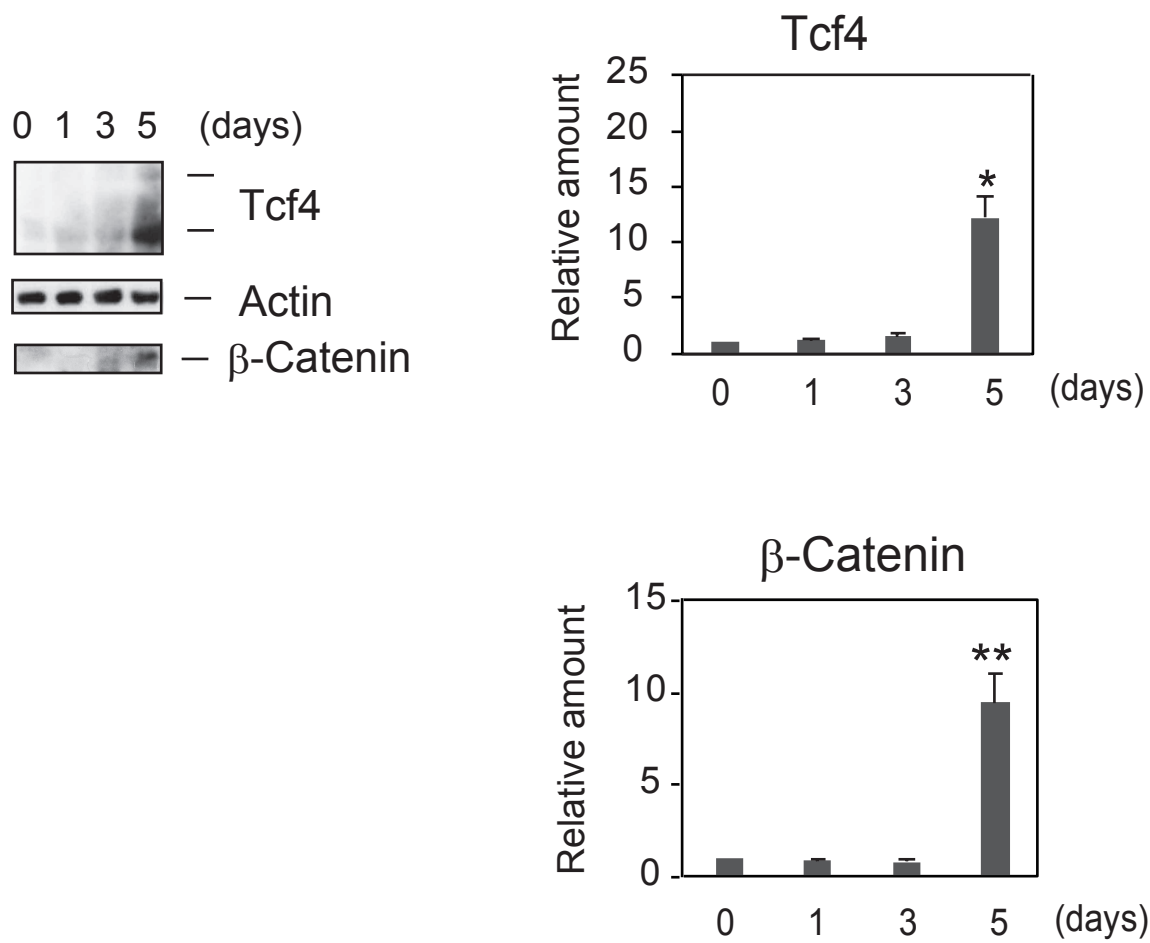

Supplement: Supplementary file 1 [file FBA2-1-435-s001.pdf]
