# Supplementary material for: Kras promotes myeloid differentiation through Wnt/β‐catenin signaling
Source: FASEB Bioadv. 2019 Jul 1;1(7):435–49. doi: 10.1096/fba.2019-00004 (PMC6996383; doi:10.1096/fba.2019-00004)
Supplement: Supplementary file 2 [file FBA2-1-435-s002.pdf]

Manuscript: 2019-00004-T

Supplemental data

Supplemental Figure S1.

Amount of Kras is unchanged during DMSO-induced differentiation of HL-60 cells.

Cell lysates from HL-60 and DHL-60 cells were subjected to SDS-PAGE and western blotting with anti-Kras or anti-actin antibody. Kras levels in lysates were calculated relative to the amount in HL-60 cells at time (t=0) and are reported as the mean ± S.E. of six independent experiments.

Supplemental Figure S2

Knock down of Kras does not effect CD11 b expression in HL-60 cells.

HL-60 cells were transfected with Kras or control siRNA (100 nM) and cultured in RPMI medium without DMSO. After 4 days, the cells were harvested and CD11b expression was analyzed by flow cytometry. In separate experiments, the expression of CD11b in DHL-60 cells (cultured for 4 days in medium containing 1.3% DMSO) was analyzed by flow cytometry. The results shown are representative of four independent experiments.

Supplemental Figure S3.

PI3K is involved in the differentiation of HL-60 cells.

(A) HL-60 cells were cultured in RPMI medium containing 1.3% DMSO plus either LY294002 (PI3K inhibitor) or LY303511 (negative control). After 5 days, the cells were lysed and the p-GSK3β/GSK3β ratios analyzed by immunoblotting. The blot shown is representative of three independent experiments (left panel). The quantified results are the means ± S.E. of three independent experiments. *$p< 0.05$. (B) In a separate experiment, cells were harvested and CD11b expression analyzed by flow cytometry. A typical flow chart is shown in the left hand panel. The ratio of the geometrical mean of CD11b expression on the cells treated with LY294002 to that on the cells treated with LY303511 is shown as the mean ± S.E. of three independent experiments (right panel). *$p<0.05$.

Supplemental Figure S4.

Wnt-related molecules are enhanced during DMSO-induced differentiation of HL-60 cells.

Lysates of HL-60 and DHL-60 cells were analyzed by western blotting with specific

antibodies targeting the molecules indicated, with the results shown being representative of four independent experiments (upper left panel). The time course of the expression of Tcf4 (upper right panel) and β-catenin (lower right panel) in the differentiated cells relative to resting cells (HL-60 cells at t=0) are shown as the means $\pm$ S.E. of four independent experiments. *$p < 0.05$, **$p < 0.01$.
